# Neural cell diversity in the light of single-cell transcriptomics

**DOI:** 10.1080/21541264.2023.2295044

**Published:** 2024-01-23

**Authors:** Sandra María Fernández-Moya, Akshay Jaya Ganesh, Mireya Plass

**Affiliations:** a Gene Regulation of Cell Identity, Regenerative Medicine Program, Bellvitge Institute for Biomedical Research (IDIBELL), Barcelona, L’Hospitalet del Llobregat, Spain; b Program for Advancing Clinical Translation of Regenerative Medicine of Catalonia, P- CMR[C], Barcelona, L’Hospitalet del Llobregat, Spain; c Center for Networked Biomedical Research on Bioengineering, Biomaterials and Nanomedicine (CIBER-BBN), Madrid, Spain

**Keywords:** Cell diversity, single-cell transcriptomics, neural cells, brain, gene regulatory networks

## Abstract

The development of highly parallel and affordable high-throughput single-cell transcriptomics technologies has revolutionized our understanding of brain complexity. These methods have been used to build cellular maps of the brain, its different regions, and catalog the diversity of cells in each of them during development, aging and even in disease. Now we know that cellular diversity is way beyond what was previously thought. Single-cell transcriptomics analyses have revealed that cell types previously considered homogeneous based on imaging techniques differ depending on several factors including sex, age and location within the brain. The expression profiles of these cells have also been exploited to understand which are the regulatory programs behind cellular diversity and decipher the transcriptional pathways driving them. In this review, we summarize how single-cell transcriptomics have changed our view on the cellular diversity in the human brain, and how it could impact the way we study neurodegenerative diseases. Moreover, we describe the new computational approaches that can be used to study cellular differentiation and gain insight into the functions of individual cell populations under different conditions and their alterations in disease.

## Introduction

In the last decade, new technologies that allow characterizing the transcriptomic make up of individual cells have arisen. Collectively called single-cell transcriptomics, or single-cell RNA-seq (scRNA-seq), these methods have rapidly evolved from manually processing one or a few cells per experiment to allow measuring the expression of hundreds or a few thousand genes for each cell for a few thousands of cells in a single experiment (reviewed in [1,2]) (Box 1). There are plenty of different technologies to perform single-cell transcriptomics (reviewed in [1,3]). Nowadays, most popular methods such as 10X genomics use droplet-based microfluidics technologies to encapsulate and tag the 3’ends of polyadenylated RNAs from individual cells. Other technologies using nanowells [4–6], or plates [7–10] are also garnering more attention as they allow higher cell throughput and easy sample multiplexing in a single experiment. However, the choice of single-cell technology is non-trivial, depending on multiple factors such as availability of cells, the biological question posed, the sample preparation, and the cost of sequencing. For instance, if the cell population of interest is rare or hard to obtain (less than 5000 cells), droplet-based technologies will not be an option as they require many more cells as input (minimum amount around 50,000 cells) and only some plate-based methods such as Smart-seq2/Smart-seq3 or Cel-seq2 would be an option (see for instance [3]). Similarly, the number of cells and the sequencing depth have to be chosen considering the scientific question and the characteristics of the sample. While commonly used lab cell lines such as HEK293T or HeLa have lots of RNAs, primary cells or cells that are difficult to dissociate have fewer RNA molecules and thus will need fewer reads per cell to reach sequencing saturation. Thus, there is no one-size-fits-all approach when talking about single-cell transcriptomics, and multiple factors have to be considered before choosing the right technology and sequencing strategy.

Box 1. Basic single-cell transcriptomics workflow.

**Figure:**
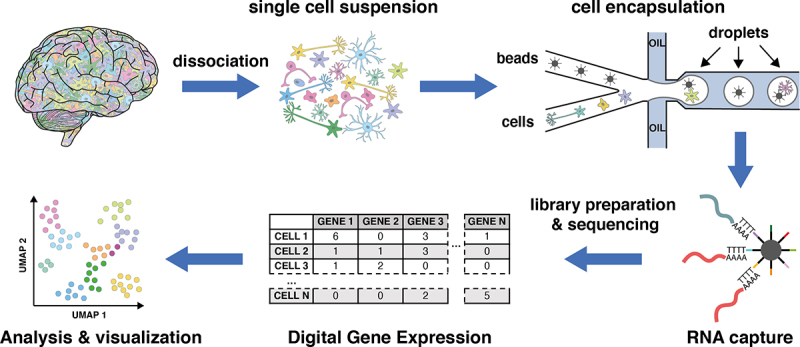
In a typical single-cell workflow, dissociated cells are first individualized in droplets or (micro)wells using microfluidics or other devices. Subsequently, the polyadenylated RNAs from each cell are captured using special oligodT primers that contain barcodes that allow to distinguish the polyadenylated RNAs that come from individual cells as well as mark the unique RNA molecules captured within each cell. At present, multiple technologies exist that have optimized this workflow to increase the throughput and the capture efficiency of RNAs (reviewed in [11]). After sequencing, computational processing of the data will quantify all the genes expressed in individual cells. With this data, we can now group cells according to their transcriptomic profiles into clusters and visually represent them using UMAPs or similar plots to study the differences that exist between them.

Apart from scRNA-seq, new methods have been developed that allow capturing other layers of cellular complexity, such as the genome and the epigenome [12]. In fact, now it is possible not only to profile the expression of genes in individual cells, but also to combine this information with that of open chromatin regions, DNA methylation, epigenetic marks, and even targeted protein expression (reviewed in [13,14]). These multimodal datasets have allowed us to better study how gene regulation occurs in individual cells and define the peculiarities that distinguish cell types and states at the molecular level. With this information, researchers are improving the definition and classification of cell types beyond morphological traits and the expression of a few surface markers.

In this review, we present how our knowledge about the diversity of cell types that compose the human nervous system has expanded with the development of single-cell transcriptomics technologies. First, we summarize some of the main atlas initiatives that have contributed to cataloging different cell populations in the human brain. Subsequently, we focus on the main neural cell types (neurons, astrocytes, oligodendrocytes, and microglia) to give an overview of our current knowledge and the main discoveries achieved with single-cell transcriptomics. Finally, we provide a brief summary of the computational methods available to study transcriptional programs in individual cells, and their contribution to study cell identity and differentiation processes.

## A new catalog of neural cells

One of the main efforts of single-cell transcriptomics technologies has been to catalog all the cell types from an organism and understand how these cells change between species, tissues, during development, and in disease. In the case of the human brain, this task has been especially challenging due to limited access to the tissue and the difficulty of dissociating adult brain cells. Thus, in almost all cases, characterization of brain cells has been done using single-nuclei RNA-seq (snRNA-seq). This technology is very similar to scRNA-seq but enables transcriptional profiling of cell nuclei [15]. Nuclei are easily obtained from cells, regardless of their tissue of origin and the condition of the sample. This approach allows the use of a wide variety of fresh, frozen and even fixed postmortem samples, simplifying the logistics. Recent studies have shown that snRNA-seq provides enough transcriptional information to define cell types with a resolution similar to scRNA-seq, despite profiling different RNA species [16,17]. Moreover, snRNA-seq provides an unbiased sampling of the cells from a tissue, as nuclei isolation is independent from the cell of origin and therefore prevents losing hard-to-dissociate cells such as neurons or oligodendrocytes [18,19]. Yet, cell nuclei contain less RNA and have significant biases toward particular RNA species that are enriched in nuclei such as pre-mRNAs [17,19], which makes the direct comparison of snRNA-seq data with scRNA-seq data harder.

Over the past few years, sc/snRNA-seq studies from multiple brain regions and time points have been used to generate detailed human brain atlases. These atlases are powerful resources for mapping the cell type heterogeneity present in the human brain as well as to characterize cellular gene expression profiles across different brain regions [20–25]. There have been several efforts to construct detailed and easy-to-access single-cell brain atlases. The first one of them was the Allen Brain Atlas. Created in 2012 [26], it started incorporating single-cell data with the release of the Allen Mouse Brain Cell Types dataset in 2018 [27]. Since then, the database has continuously been updated incorporating new single-cell transcriptomics datasets [28]. Another of such initiative is the Human Cell Atlas (HCA). The HCA started in 2016 as an international collaborative initiative aiming to create a detailed map of all human cells, including those in the brain [29]. The Brain Initiative Cell Census Network [20], which is part of the Brain Research through Advancing Innovative Neurotechnologies (BRAIN) initiative, is yet another project to catalog all cells in human, monkey, and mouse brains. Their goal also includes describing the molecular basis of brain function and its alterations in neurological and neurodegenerative diseases. In 2021, the BRAIN initiative published a cell census and atlas of the primary motor cortex through a coordinated multi-laboratory and multi-platform approach [11,30] and compared the differences across mouse, marmoset, and human samples [31].

All these atlases, along with recently published (or under revision) human brain single-cell papers [32], are facilitating the identification and classification of cell types in the brain, providing an unprecedented view of the neuronal diversity landscape, and shedding light on their lineage relationships, cell states and dynamics during development, aging, and disease.

## Brain cell type diversity

The brain –as well as other organs in our body– was believed to consist of a limited repertoire of cell types, primarily neurons, and some well-characterized glial cell types. However, the analysis of single-cell transcriptomics data from different brain regions and across development has led to the identification of previously unrecognized subclasses of cells and states, revolutionizing our view of cellular diversity within the brain. By analyzing the transcriptomic profiles of individual cells, it has been possible to tease apart the molecular signatures and functional characteristics of each of these new neuronal subpopulations [22,33]. Below we highlight the most recent works published and analyze the latest discoveries about cellular and molecular diversity in the adult human brain.

## Neuronal complexity

**Figure 1.**
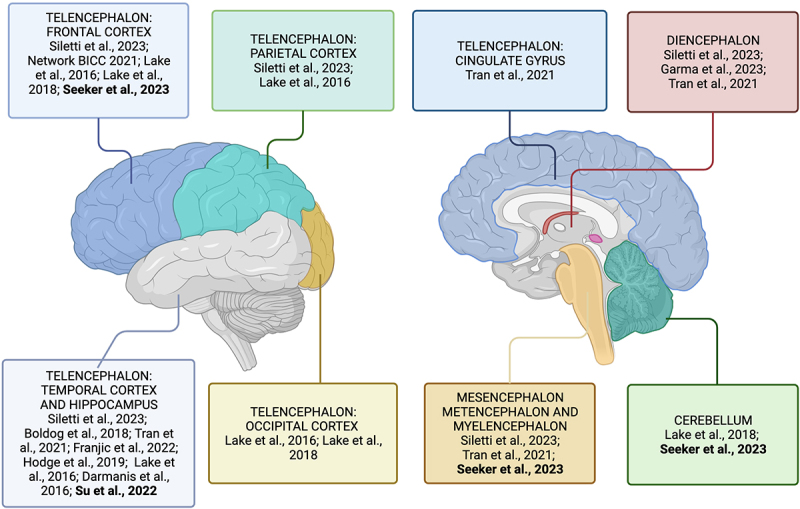
snRNA-seq studies reveal the cellular complexity of the adult brain.

Single-cell sequencing studies have revealed a plethora of unknown neuronal cell types within the brain, showing intriguing differences in neuronal diversity between brain regions. Up till now we can cover the neuronal diversity in the human brain with several important studies from the cortex [22,33,34], hippocampus [35], striatum [25,36], midbrain [37,38], cerebellum [22], hypothalamus [39], and even retina [40,41] (Figure 1). Several reviews have been recently published describing the importance of sc/snRNA-seq for the analysis of the transcriptomic profiles and the compositional changes in cell populations during development [42] and upon disease [43]. Therefore, we will mainly focus on the most recent works focused on postnatal human brain samples.

snRNA-seq studies in the adult human brain reveal a complex neuronal and glial heterogeneity at cellular and molecular levels within and across different regions of the brain. The highlighted papers focused their work on a deeper analysis of glial cell populations in the brain.

## Cortical neurons

Neurons are the key feature of cortical circuits where, in response to a specific stimulus, they receive and process signals, and transmit their response to other neurons downstream in the signaling current. Cortical neurons can be divided into two major types regarding their main output signal: excitatory glutamatergic neurons and inhibitory GABAergic interneurons. Interneurons enable a proper inhibitory pathway working together with excitatory neurons to conduct complex and fine motor movements as well as fine sensitivity [44]. Failure in the function of either excitatory or inhibitory neurons is associated with neurodegenerative, neurodevelopmental, and psychiatric disorders such as autism spectrum disorder (ASD), epilepsy and schizophrenia [45,46].

The diversity within excitatory and inhibitory neuron populations was first identified based on their localization in the different cortical layers (L) and brain regions. Each brain region is associated with a specific function (sensory, motor, or associative) and each function depends on its connection to other structures and regions of the brain. For instance, the primary visual cortex is located in the occipital cortex, the most caudal part of the telencephalon. This region is involved in the preprocessing and analysis of information received through the retina, which is transported to the lateral geniculate nucleus and subsequently, to the thalamus and the primary visual cortex. By contrast, the primary motor cortex is located in the frontal cortex region and controls body movement through its connection to the spinal cord and the muscles. While different brain regions are associated with specific functions, the role and diversity of neurons in different cortical layers is not so well defined. Thus, the identification and categorization of these neurons is an essential step to understand cortical circuits.

One of the first studies that investigated the complexity of the adult brain at the single-cell level analyzed fresh adult human temporal lobe samples from living subjects who underwent temporal lobectomy [47]. Although this work only included 466 cells, far below the standards of current technologies, the authors identified all typical cell types from the brain, including oligodendrocytes, oligodendrocyte precursor cells (OPCs), astrocytes, microglia, endothelial cells and neurons. Interestingly, this work already identified significant discrepancies in the expression of genes between human and mouse. For instance, the authors observed that most inhibitory neuron populations expressed cholecystokinin (CCK) and calbindin 2 (CALB2), while only a small fraction of them expressed parvalbumin (PVALB). This result differed from previous analyses in mice reporting that CCK is expressed only in a small subset of inhibitory neurons, while 40% of them are PVALB positive [48]. Darmaris and coauthors also showed that interneurons in L1 express markers such as reelin (RELN) and paired box protein 6 (PAX6) typically considered markers of radial glia and progenitor cells in the developing cortex. Another surprising result was the identification of major histocompatibility complex class I (MHC-I) genes, primarily related to immune responses or pathological conditions, in a subset of neurons in the adult brain. These neurons expressed MHC-I genes at a level similar to that of endothelial cells and microglia, indicating that MHC-I proteins are indeed important for brain activity [49]. This expression was not found in fetal brain, which suggests a postnatal onset of MHC-I expression in human brain.

Later studies such as Lake et al. [15] used snRNA-seq to comprehensively profile neuronal diversity across six Brodmann Areas (BA) corresponding to frontal (BA 8 and 10), temporal (BA 21, 22, 41 and 42) and occipital visual (BA 17) cortex. To obtain neuronal transcriptomic profiles, the authors used fluorescence-activated cell sorting to isolate the nuclei of cells expressing the neuronal nuclear antigen (NeuN), which marks mature neurons. After sorting, single nuclei were processed using Fluidigm C1 and sequenced. The neuronal populations identified were strongly related to their anatomical position in the brain. Mostly excitatory neurons but also some inhibitory neurons showed differences at the gene expression level associated to the brain region where they were located. Interestingly, the higher complexity of cortical layers in the visual cortex (L4, which receives most visual input, is further divided into 4 sublayers) was also reflected in the cell populations identified, as these layers contain the largest number of excitatory and inhibitory cell types identified. For example, inhibitory neuron populations within L3 in BA 17 showed a specific absence of RELN/somatostatin (SST) expression, which is found in a specific subtype of L3 inhibitory neurons in other cortical regions, reflecting the intricate molecular diversity within the cortex and its potential implications for cortical circuitry and function.

Additional studies have also been performed to understand the cellular composition and organization within specific neocortical circuits. Boldog and coauthors [50] studied the diversity of inhibitory neurons within L1 of the middle temporal gyrus. L1 is interesting as recent studies suggest that this layer could be crucial for long-term plasticity and associative learning through a connection to the hippocampus [51]. This work identified a new type of interneuron. These neurons, which were called rosehip neurons, are characterized by large rosehip-like axonal boutons and display a unique gene expression profile including glutamate decarboxylase I (GAD1) and CCK genes while lacking the expression of cannabinoid receptor 1 (CNR1), SST, CALB2, and PVALB. Functional analysis of these cells showed that they connect to apical dendritic shafts of L3 pyramidal neurons, suggesting that rosehip neurons locally control pyramidal actions in microdomains of the cortex [50].

Aiming to describe the full diversity of neuronal cell types in the brain, the most recent work categorizing cortical neurons used three postmortem donors to analyze the transcriptomic diversity from around 100 dissections across of the telencephalon, diencephalon, midbrain, and hindbrain [32]. This work shows the presence of 21 neuron superclusters in the brain. In agreement with previous works [15], they also found the presence of region-specific gene expression changes within each of these clusters. Interestingly, the authors found that layer-specific excitatory neurons varied more across dissections than layer-specific inhibitory neurons. Inhibitory neuron cell clusters often contained cells located in different cortical regions. In contrast, some excitatory neuron clusters were enriched in cells from specific brain regions (e.g., visual cortex, anterior cingulate cortex, or entorhinal cortex), which are areas of high functional specialization. These results suggest a unique mode of brain activity regulation based on the specialization of excitatory neurons in different regions and the regulation of these neurons through a vast network of inhibitory neurons that connect different brain regions, coordinating, and transmitting information across longer distances.

Together, the study of cortical neurons at the single-cell level has revealed a complex and diverse landscape within the human brain. The presence of region and layer-specific neuronal populations highlights the close link between cellular diversity and cortical function. For example, the fact that a unique interneuron type like rosehip neurons [50], which are involved in signal regulation of upper cortical neurons, has been identified only recently, demonstrates the potential of these studies to understand cortical circuits and their functions.

## Non-cortical neurons

The study of neuronal diversity within key brain regions associated with neurodegenerative diseases such as the striatum, the hippocampus, and the amygdala has uncovered distinct sets of populations of neurons, including dopaminergic medium spiny neurons (MSNs). So far, these works have found clear relationships between cell types and brain function. Yet, it is likely that many rare cell populations that participate in all these processes remain undiscovered. Thus, a thorough study of region-specific cellular diversity at different developmental stages and in adulthood will be needed to better understand brain function and develop treatments against neurodegenerative diseases.

### Striatum

There is a special focus on the analysis of neuronal diversity within the striatum and hippocampus due to their involvement in neurodegenerative diseases such as Parkinson’s (PD) and Alzheimer’s Disease (AD) [43]. The striatum is the main entrance to the whole basal ganglia system and is mainly formed by GABAergic neurons that locally fine-tune the signal received from the cortex before sending it onward to other basal ganglia nuclei. The majority of striatal neurons are the so-called MSNs, multipolar neurons that possess multiple dendrites densely covered by spines as well as an axon [52]. MSNs are GABAergic neurons characterized by the expression of dopaminergic receptors, which defines the existence of two types of MSNs: D1-type, which express D1 dopaminergic receptor, and D2-type, which express the D2 receptor [53]. Aiming for a deep characterization of the diversity of neurons that form the human reward circuitry, Tran et al. [25] profiled the ventral striatum, hippocampus, nucleus accumbens, amygdala, subgenual anterior cingulate cortex, and dorsolateral prefrontal cortex using snRNA-seq. The authors confirmed that the nucleus accumbens, which plays a role in motivation and cognitive processing of aversion, is mainly formed by MSNs. MSNs can be classified in 10 different subtypes, including a set of MSNs expressing both D1 and D2 dopamine receptors. This neuronal subset was part of a larger neuronal population characterized by the expression of genes associated with psychiatric and substance use phenotypes, suggesting that this population may be involved in these disorders. Interestingly, the authors found a promiscuous expression of typical D1 and D2 MSNs markers, indicating that these classic markers may not be as unique as previously described. For instance, tachykinin precursor 1 (TAC1), a neuropeptide characteristic of D1 MSNs [54], was enriched in specific D2-type MSNs populations and absent in some D1-type MSNs subtypes. Similarly, proenkephalin (PENK) [55], which was a known marker of D2 MSNs, was absent in some of the smaller D2-type cell populations. Together, these results highlight the importance of sc/snRNA-seq experiments in the identification and characterization of neural populations beyond immunohistochemistry analyses.

Up to now, the molecular characteristics of other types of striatal interneurons have not been properly analyzed. To elucidate their diversity and abundance, Garma et al. [56] analyzed almost 20,000 interneuron nuclei from the caudate nucleus and the putamen. The authors defined eight main classes of interneurons that are not MSNs and named them after their main molecular markers: CCK/vasoactive intestinal peptide (VIP), CCK, PVALB, SST/glutamate ionotropic receptor kainate type subunit 3, GRIK3, SST/neuropeptide Y (NPY), parathyroid hormone like hormone (PTHLH), choline o-acetyltransferase (CHAT) and tachykinin precursor 3 (TAC3). A novel interneuron population only expressing PTHLH was identified as one of the most abundant interneuron classes together with TAC3 interneurons. This population was previously identified [25] as a unique interneuron cluster coexpressing PVALB/PTHLH, which was confirmed in mice [36]. Interestingly, the amount of PVALB+/PTHLH- and PVALB-/PTHLH+ interneuron populations was highly variable across donors, while the number of PVALB+/PTHLH+ cells (as seen in mouse) was consistent but more rare. Some of the clusters defined by Garma and coauthors were also previously identified [25] with small differences: SST and NPY-expressing clusters were defined as two independent ones instead as a single one; the VIP cluster was also identified as a single cluster; and the TAC3 cluster was found to be shared with mouse, indicating that some inhibitory neurons are present in both species and could be of relevance for the study of basal ganglia pathologies.

### Hippocampal cell populations

The hippocampus, as the key structure for long-term and short-term memory formation and consolidation, has been exhaustively characterized in mice using single-cell transcriptomics (reviewed in [35]). Anatomically, the hippocampal formation has three main parts: the hippocampus or cornu ammonis (CA) which is divided in 4 regions (CA1–4), the dentate gyrus (DG), and the subiculum. Additionally, the hippocampus displays functional and structural differences along its anteroposterior axis. These differences are related to the functions controlled by the hippocampal neurons in these areas and are reflected in their expression profiles [57,58]. To understand the cellular and molecular diversity along this axis and define molecular signatures corresponding to functional domains, Ayhan and coauthors [48] performed snRNA-seq on surgically resected human anterior and posterior hippocampus from epileptic patients. They found that the excitatory DG granule cells, which are the main regulators of the flow of information in the hippocampus and are required for learning and memory formation, are highly diverse and display axis-specific gene expression signatures. Genes differentially expressed in the posterior hippocampus are related to cognitive function, such as ion channels (potassium inwardly rectifying channel subfamily J member 6, KCNJ6), synapse organization (neuronal pentraxin receptor, NPTXR, and slit guidance ligand 1, SLIT1), and cytoskeletal organization (syntaxin-binding protein 5-like, STXBP5L). In contrast, there is a higher expression of genes associated to a wide range of functions, such as mood and affection in the anterior hippocampus, finding markers for behavioral fear response (5-hydroxytryptamine receptor 2C, HTR2C), RNA binding proteins (RALY RNA binding protein like, RALYL, and KH RNA binding domain containing, signal transduction associated 3, KHDRBS3), or cell-cell adhesion (cell adhesion molecule 1, CADM1). One of the identified DG clusters was described as a precursor granule cell with potential neurogenic capacities as it expressed the neuronal stem cell marker lysophosphatidic acid receptor 1 (LPAR1). This result would support the existence of adult neurogenesis in human but would need further validation. This pattern is not seen among inhibitory neurons or other non-neuronal cell types such as astrocytes and microglia.

### Neurogenic cell populations in adult hippocampus

Hippocampal adult neurogenesis remains a controversial topic [59]. Cross-species analysis of hippocampus and entorhinal cortex between human, macaque and pig found that doublecortin (DCX)-expressing DG cells lack neurogenesis signatures in humans although these signatures were present in the other species [60]. Based on this analysis, the authors concluded that baseline neurogenesis does not occur or is extremely rare in humans. However, these results disagreed with a previous study that identified a DG granule cell population expressing LPAR1 that displayed neurogenic capacity [61].

More recently, Zhou and coauthors combined a machine learning approach with snRNA-seq to identify and quantify immature dentate granule cell (imGC) populations during human lifespan [62]. ImGCs are newly born neurons especially abundant in infant brains that prove the existence of neurogenesis. Using this approach, the authors identified stathmin 1 (STMN1) together with prospero homeobox 1 (PROX1) and DCX as markers for imGCs. The use of the trained machine learning model to identify imGCs in previously published samples [60,61] confirmed the presence of imGCs in adulthood (3.1–7.5%). Considering these results, the authors proposed a model in which new neurons in the hippocampus are derived from immature granule cells, which are maintained in this state for a long period of time, rather than from proliferating neural progenitors.

Taken together, all these works suggest that more studies, with a larger number of cells and deeper sequencing are required to detect and fully confirm neurogenic populations in humans.

### Cell type diversity in the amygdala

Another important brain region that has been profiled using single-cell transcriptomics is the amygdala [25,63]. The amygdala is an important structure in memory formation and processing of emotions and has been implicated in neuropsychiatric diseases such as bipolar disorder and posttraumatic stress disorder [64–66]. scRNA-seq data has shown that the majority of excitatory neurons from the amygdala can be grouped in a single supercluster [32], highlighting a low variability among these cells. In contrast, inhibitory neurons are divided into multiple clusters that are strictly distributed within specific amygdala regions [25,32,63]. The authors also discovered a cluster of immature excitatory neurons in the paralaminar nucleus that expressed genes characteristic of neuronal progenitors. They hypothesized that these cells could stay in latency for decades and contribute to plasticity in the reward circuitry during adulthood, although their function is not really known [63].

## Non-neuronal populations

Neuronal diversity has been the special focus of most single-cell studies on human postmortem brain samples [22,25,32,60,61,67]. Although these studies have also analyzed the non-neuronal cell populations, these have not been explored in-depth. Glial cells, which comprise around half of the cells of the adult human brain, have a key role in brain homeostasis as they work as supportive (astrocytes and oligodendrocytes) and protective (microglia) cells in the brain. New studies additionally highlight the role of glial cells in neuronal dysregulation and vulnerability associated with neuropsychiatric and neurodegenerative diseases [68]. Therefore, the systematic identification and classification of glial cell populations is needed to understand and treat brain pathologies.

### Astrocytes

Astrocytes are the most abundant glial cells in the human brain [69]. They are involved in the maintenance of the blood-brain barrier and the regulation of blood flow as well as provide support for neurons, aid neurotransmitter recycling and regulate synaptic transmission [70]. Before single-cell studies, astrocytes were considered to be the most homogeneous population in the brain. However, the heterogeneity of their shapes across brain regions led to a renewed focus on their diversity, both at the structural and functional level.

To decipher the molecular diversity of glial cells within the hippocampus and their temporal dynamics over the full lifespan, Su and coauthors [71] analyzed samples from infant, child, adolescent, adult, and aging stages of life. They identified nine astrocyte populations with different gene signatures and transcription factor (TF) expression. Each of these subpopulations was associated with different physiological functions such as angiogenesis, regeneration, or gliogenesis. Interestingly, many of these populations remained unchanged during aging. Only the glial fibrillary acidic protein (GFAP) and the SRY-box transcription factor 2 (SOX2)-enriched populations changed with age. While the number of GFAP-enriched astrocytes –which are considered reactive astrocytes– increased with aging, the number of SOX2 astrocytes –a putative progenitor cell population– decreased, similar to what had been observed within the reward circuit [18]. Moreover, these analyses also revealed an astrocyte population in the amygdala which was metabolically low-active. These astrocytes lacked the expression of central transcription factors such as SOX6, TCF4 and the nuclear factor I/A (NFIA) and signaling/growth regulators such as AKT (also known as protein kinase B, PKB) or adenomatous polyposis coli (APC). These findings suggest that this cluster could represent a resting or dormant astrocyte population, although these observations need to be further studied.

Together, these works revealed a remarkable heterogeneity among astrocytes located in different brain regions. This diversity is characterized not only by structural differences but also by distinct functional roles encoded in their gene signatures, shedding light on the multifaceted roles of astrocytes in brain function and their potential implications in neurodegenerative diseases.

### Oligodendrocytes

Glial cells such as oligodendrocytes (OLs) have been traditionally considered to be a rather homogenous population beyond their differentiation status as oligodendrocyte progenitor cells (OPCs), immature pre-myelinating oligodendrocytes (immature OL) and mature myelinating oligodendrocytes. Yet, ever since their discovery by Pío del Río Hortega over a century ago, OLs were described as cells with varying amounts of processes and localizations [72]. Recent advances in snRNA-seq have confirmed these initial observations and revealed a much broader diversity among OL cells including developmental, regional and functional diversity (reviewed in [73–75]) as well as the presence of cell populations not present in model organisms such as mouse [71], highlighting the differences in OL function across species.

To understand the physiological variation within white matter glia, several groups have done an extensive study of OLs within different brain regions considering the age and the sex of donors. There is a general consensus across publications about the OL populations identified [32,71,73–75]. Mature OL populations were characterized by the expression of the three specific OL markers: the glycosylphosphatidylinositol (GPI)-anchored protein OPALIN, the RNA-binding fox-1 homolog 1 (RBFOX1), and the secreted protein acidic and rich in cysteine (SPARC). The latter was defined as a new OL marker found only in cervical spinal cord [73]. OPALIN OL clusters were composed of cells predominantly from the telencephalon while RBFOX1 OL clusters contained cells from posterior regions of the brain and were not observed in mouse OLs [32]. OPCs and immature OL expressed genes characteristic of progenitors such as NG2 (also known as chondroitin sulfate proteoglycan 4, CSPG4) and SOX6. Yet, while immature OLs expressed genes related to myelination, the gene signatures of OPCs suggested the existence of specialized OPC populations with function-specific [71], region-specific [32] as well as age-related [73] transcriptional variation. Based on their function, OPCs can be subdivided into two main populations. The main OPC population expressed CD74, a gene related to the class II major histocompatibility complex (MHC-II), which suggests a role for this OPC population in immunomodulation [71]. In contrast, the other OPC population expressed genes related to WNT signaling, which could be related to angiogenesis regulation [76]. Depending on their location, two OPC groups were also defined. OPCs from telencephalon (expressing neural EGFL-like 1, NELL1) have a high proliferation rate, indicating a high frequency of differentiation that should help to replenish the OL populations more often in the telencephalon. These OPCs also showed different transcription profiles compared to OPCs from non-telencephalic regions (spinal cord, expressing paired box 3, PAX3) [32,73]. More than 150 genes were differentially expressed between NELL1 and PAX3 populations, including axon guidance molecules, region-specific transcriptional factors and notch signals [32].

With aging, OPCs display changes in the expression of the early myelin protein proteolipid protein 1 (PLP1) and the membrane receptor mediating proliferation protein alpha (PDGFRA), indicating a decline in OPC function with age. Aged OPCs also have lower expression of SOX6, suggesting a decrease in the proliferative function as SOX6 has been linked to the maintenance of the OPC state [77].

In conclusion, these discoveries underscore the previously underestimated complexity of oligodendrocyte populations and their crucial roles in brain development, regionality, function, and age-related changes. Further investigations promise to unveil the intricate mechanisms governing glial cell diversity and its significance in neurological health and disease.

### Microglia

Microglia are the innate immune cells of the brain. These cells similar to macrophages can shift from a homeostatic to an activated phenotype –the latter characterized by markers of proliferation, chemotaxis and changes in morphology [78]– once they encounter damage in the brain. Microglial cells are also important in neurodegenerative diseases such as AD and PD [79,80]. Several single-cell studies demonstrate that microglia differ in their abundance, regional localization, and transcriptomic profiles across the brain ([71,79,81] and reviewed in [82,83]).

The most abundant microglia population is formed by cells expressing markers for the homeostatic phenotype (purinergic receptor P2RY13 and the C-X3-C motif chemokine receptor 1, CX3CR1) and immune surveillance (CD83, early growth response 3, EGR3, and C-C motif chemokine ligand 2, CCL2) [79,81]. A second population of microglia identified, which expresses secretin phosphoprotein 1 (SPP1) and the triggering receptor expressed in myeloid cells 2 (TREM2) markers, is related to autophagy and neuroinflammatory response, previously reported to be disease-associated [84]. The remaining populations express classical neuronal or glia genes such as those related with axon and synaptic modulation (CUB and sushi multiple domains 1, CSMD1), myelination, and glia differentiation (expressing myosin 1d, MYO1D, and plakophilin 4, PKP4) [71,73].

Abundance of microglial cell populations changes with age and some subtypes appear exclusively after puberty or in aging [71,73,79,84]. For instance, homeostatic and immune surveillance populations increase with age [71] and are absent in infant and child tissue. Another microglial population exclusive from adult brains is characterized by the expression of interferon response signaling pathway genes (interferon regulatory factor 1, IRF1, and 10, IRF10) and genes related to MHC-II antigen presentation such as the ETS variant transcription factor 4 (also called PEA3) or the zinc finger E-box binding homeobox 1 (ZEB) [71,73,79,84]. In contrast, neuroinflammatory-related microglia decrease with age [71].

Furthermore, there are also sex-specific differences in the expression of pro-inflammatory genes in microglia such as the MHC-II, DR beta 5 protein (HLA-DRB5) and the interferon induced protein 44-like (IFI44L), which are genes more commonly expressed in aging women. This indicates that both aging and sex are to be taken into consideration as susceptibility factors for inflammatory responses in the brain [71].

The diversity of microglia populations and their dynamic responses according to age and sex underscore the complexity of their role in brain health and disease. Further investigations will be crucial to unravel the intricate mechanisms governing microglia diversity and their impact on neuroinflammatory processes.

## Studying transcriptional programs at the single-cell level

**Figure 2.**
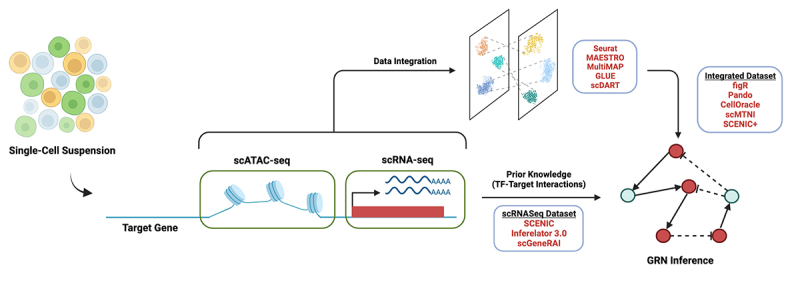
Gene regulatory network inference from single-cell omics data.

Apart from identifying cell type diversity, single-cell transcriptomics methods have been used to study the transcriptional programs that characterize cell types and drive cell differentiation. Several computational tools (for example see [28,85–90]) have been developed that allow defining Gene Regulatory Networks (GRNs) at the single-cell level, that is, the set of TFs that interact with each other and regulate target genes to perform a specific function (Figure 2). Given that TFs are the main drivers of gene expression and thus largely responsible for the functional differences among cells, identifying and characterizing GRNs is essential to understand cellular diversity.

The simplest methods use scRNA-seq to measure gene expression in individual cells and use this data to identify groups of coregulated genes, which will likely be regulated by the same TFs. In some cases, these methods use prior knowledge such as previously known TF binding sites to obtain more reliable GRNs. Combining scATAC-seq, which provides regulatory element information, and scRNA-seq as a read-out of gene expression is a more robust approach to infer GRNs. Computational integration of scRNA-seq and scATAC-seq datasets and joint cluster analysis can be performed to identify cell populations and better define cell-type-specific GRNs.

To define GRNs, the goal of these methods is to identify TF-target gene relations across multiple cells using single-cell data. However, this is non-trivial as there are multiple confounding factors in single-cell data including *dropouts*, variation in the sequencing depth of each cell, and batch effects, among others [91]. Moreover, it has to be considered that gene expression is used as a proxy for TF abundance and activity, although in many cases this assumption is not correct [92]. Some of the most successful methods at predicting GRNs, such as SCENIC [86,87] or the Inferelator 3.0 [89] are those that take advantage of prior information of TF binding across the genome. This information can be extracted from high-throughput chromatin immunoprecipitation (ChIP) assays coupled to sequencing (ChIP-seq) experiments [93], which provide experimental knowledge of the binding of a specific TF across the genome, or computational prediction of TF binding sites that can be extracted from databases such as TRANSFAC [94] or JASPAR [95]. This TF binding site information is then used to filter spurious correlations between TF and target genes and restrict the analysis to known TF-target gene pairs. This approach can partly compensate for the presence of dropouts when inferring correlations from single-cell data.

More recent methods take advantage of combining scRNA-seq data with another single-cell omics technology: scATAC-seq data. The assay for transposase-accessible chromatin using sequencing (ATAC-seq) technology was developed a decade ago [96] as a method to identify open chromatin regions, which usually represent regulatory elements in the genome such as promoters and enhancers [97]. This technology was rapidly adapted to profile open chromatin regions in single cells [97–99]. As in bulk, scATAC-seq has been used to unbiasedly identify regulatory elements in the genome that can be used to identify cell populations [100–104]. Motif analysis in these regions leads to the identification of TFs that regulate the expression of nearby genes, also allowing to reconstruct GRNs. The problem with scATAC-seq data is that open chromatin regions do not always reflect active regulatory elements or elements that drive transcription of nearby genes, which complicates the construction of GRNs. Therefore, the combination of scATAC-seq, which allows the identification of the regulatory regions in the genome, with scRNA-seq data that provides the readout of gene expression in individual cells, provides the most robust method for the identification of GRNs.

Several computational methods have been developed to combine scRNA-seq and scATAC- seq data from different samples [101,105–111]. In this case, scATAC-seq data is used as a readout of gene expression, which is then used to integrate scRNA-seq and scATAC-seq datasets to jointly perform clustering analysis. In this way, we can define the genes expressed in a particular cell population (cell cluster) and its regulatory regions. Newer multiomics technologies now allow obtaining both transcriptomic and open chromatin readout of the same cells [112–116]. These computational methods have also been summarized in Figure 2. Working with these data, we can directly use software to infer GRNs combining transcriptomic readouts and open chromatin regions [106,117–120] without having to infer gene expression or active regulatory elements.

## Reconstruction of cellular trajectories and cell differentiation

**Figure 3.**
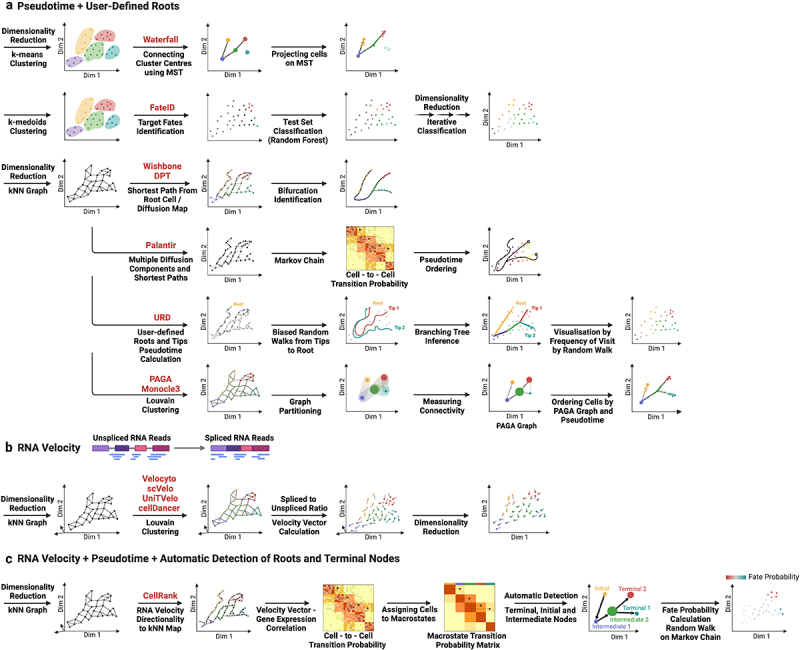
Computational strategies to reconstruct cellular lineages from single-cell transcriptomics data. (a) computational methods for linear reconstruction that use pseudotemporal ordering of cells based on the comparison of individual transcriptomic profiles. Most of these methods require the definition by the user of a “root” cell from which to compute the pseudotime. (b) RNA velocity-based methods use reads mapped to spliced and unspliced RNAs as a proxy to calculate the future transcriptomic profile of a cell. These future states can be used to define the individual cell trajectories. (c) Newer models such as CellRank combine the kNN graph to define trajectories and RNA velocity approaches to determine their directionality in a probabilistic manner, allowing the user to define initial, intermediate and terminal differentiation states.

scRNA-seq has also amply been used to infer cell differentiation trajectories and study the genes driving these processes (reviewed in [121], Figure 3). Here, the main assumption is that cell differentiation is a gradual process that is reflected in the gene expression profile of cells. If this is true, then any set of cells can be ordered according to their transcriptomic similarity to define a differentiation process or trajectory. However, this is easier said than done as differentiation trajectories are complex and single-cell transcriptomics methods do not provide unbiased sampling of differentiation processes.

The first programs developed to study cell differentiation supported simple scenarios assuming linear [122,123] or bifurcating [124,125] trajectories. In some methods, clustering of cells according to their transcriptomic profiles was used to define cell populations and project the cells from these clusters into a linear trajectory [123]. Other methods used the transcriptomic profiles of cells to build a graph connecting cells according to their similarity (a k-nearest neighbour or kNN graph) and project the cells into a single trajectory [122,123] or bifurcating trajectories [124,125] (Figure 3a). While the kNN graph approach was an interesting way of defining cell similarity without the constraints of classical clustering approaches, the applicability of these methods was very limited as they could only be used to order cells in a single-trajectory, i.e., in the case of the differentiation of stem cells to a particular cell type *in vitro*, or sorting cells where a progenitor cell type gives rise to two different cell populations, as for instance CD4+ and CD8+ T cells that come from lymphoid progenitors [126]. Using kNN graphs, more complex algorithms have been developed that are able to infer complex trajectories from the single-cell data including trees, loops, and even the presence of independent trajectories [105,127,128] (Figure 3a). These methods have been successfully applied to study developmental trajectories during zebrafish development [127] and mouse organogenesis [129], cellular trajectories of planarian flatworms [130], and even functional alterations in the brain in neurodegenerative diseases such as PD [131] and AD [132]. The main drawback of these methods is that they require prior information to define the initial cell state from which to define the pseudotemporal ordering, which limits its usage to processes for which that information is available.

An entirely different set of methods has been developed based on the concept of RNA velocity (Figure 3b) [133]. Rather than defining differentiation trajectories, these methods infer the future transcriptomic state of individual cells by modeling the reads coming from spliced and unspliced RNAs from the same gene [133–136]. These future states can be used to define the differentiation trajectories of individual cells independently, which can be later projected on a low dimensional space such as UMAP or tSNE plot to get an understanding of the relationships between the cells in a dataset.

Newer and more powerful methods such as CellRank combine these two previous approaches in a probabilistic manner to define cellular trajectories [137] (Figure 3c). CellRank incorporates probabilistic pseudotime ordering and RNA Velocity to perform lineage tracing and define initial, terminal and intermediate cellular states in the lineage without prior user specification. Thus, it can be used to investigate previously undefined differentiation trajectories under normal conditions as well as during regeneration and disease. Additionally, it provides the probability of each cell to become any of the final cellular states and therefore provides finer resolution to investigate which genes influence cell fate decisions. A summary of the main strategies used for lineage reconstruction and the main steps of the different algorithms is shown in Figure 3.

## Conclusions and future perspectives

In this review, we have provided an overview of how recently developed single-cell transcriptomics methods have increased our knowledge about the complexity and cellular diversity of the human brain. Additionally, we have provided a brief summary about how these datasets have been leveraged by new computational tools to investigate cell-type-specific transcriptional programs and gain insight into how neural fate determination and cellular diversity is achieved.

While single-cell transcriptomics has provided valuable insights on the cell diversity within the brain, there are still significant gaps in our current knowledge. Some of the open questions relate to the diversity of cell types across individuals and species. Is the cellular diversity, or even the cell composition of the brain, the same across individuals? What are the differences across species? How are they related to functional changes or even disease predisposition?

As the field continues to advance, addressing these challenges will deepen our understanding of the relation between structures and functions and their changes during evolution and in disease. Yet, some of these limitations will only be tackled with the development of newer technologies. For instance, snRNA-seq is the main method employed to analyze the adult brain at single-cell resolution given that it is impossible to dissociate intact adult brain cells. Considering that snRNA-seq only captures RNA molecules enriched in nuclei and somas, there is a lack of information about the expression of genes in specific cytosolic compartments such as the neuropil, which hinders the study of essential neural functions such as synaptic signaling and its alterations in neuropsychiatric disorders. Additionally, structural and functional insights into the roles of identified cell types and their interactions remain limited, as these technologies do not retain information about cellular location or interactions.

The single-cell omics revolution has just started, with new methods constantly being developed to address these issues and obtain more information of individual cells including protein expression, DNA methylation, epigenetic marks and genomic mutations among others (reviewed in [14,138,139]), and even determine their spatial distribution within a tissue (reviewed in [14,140]). These technologies already have and will [141] continue to have a significant impact on how we study cellular diversity in a wide range of contexts, including disease diagnostics, drug testing and development of new therapeutic approaches.

During the revision of this manuscript two special issues focused on the work of the BRAIN Initiative Cell Census Network Consortium were published on Science and Science Advances [141,142,143], and their work has not been discussed in this manuscript.
